# Improved RRT* Path-Planning Algorithm Based on the Clothoid Curve for a Mobile Robot Under Kinematic Constraints

**DOI:** 10.3390/s24237812

**Published:** 2024-12-06

**Authors:** Kemeng Ran, Yujun Wang, Can Fang, Qisen Chai, Xingxiang Dong, Guohui Liu

**Affiliations:** College of Computer and Information Science, Southwest University, Chongqing 400715, China; rkm666@email.swu.edu.cn (K.R.); wangyjun@swu.edu.cn (Y.W.); cqsllyt@email.swu.edu.cn (Q.C.); d13538@email.swu.edu.cn (X.D.); liu914904@email.swu.edu.cn (G.L.)

**Keywords:** path planning, RRT*, Clothoid curve, obstacle avoidance

## Abstract

In this paper, we propose an algorithm based on the Rapidly-exploring Random Trees* (RRT*) algorithm for the path planning of mobile robots under kinematic constraints, aiming to generate efficient and smooth paths quickly. Compared to other algorithms, the main contributions of our proposed algorithm are as follows: First, we introduce a bidirectional expansion strategy that quickly identifies a direct path to the goal point in a short time. Second, a node reconnection strategy is used to eliminate unnecessary nodes, thereby reducing the path length and saving memory. Third, a path deformation strategy based on the Clothoid curve is devised to enhance obstacle avoidance and path-planning capability, ensuring collision-free paths that comply with the kinematic constraints of mobile robots. Simulation results demonstrate that our algorithm is simpler, more computationally efficient, expedites pathfinding, achieves higher success rates, and produces smoother paths compared to existing algorithms.

## 1. Introduction

Robot path planning [1,2,3] refers to finding a feasible path from a starting point to a goal point within a given environment, enabling the robot to navigate around obstacles, comply with kinematic constraints, and reach the destination. These algorithms contribute significantly to the advancement of robotics and serve as a reference for related fields such as robot learning, robot vision, and robot collaboration. Path-planning algorithms enable study of high-dimensional spaces and non-holonomic constraint problems, applicable in scenarios like map navigation, robot operations, drone path planning, and gaming. As robot technology advances and application scenarios expand, path-planning techniques become increasingly crucial. Research in robot path planning not only enhances our understanding of robot motion patterns and control strategies across different environments but also improves robot autonomy and adaptability. This enables robots to play roles in diverse fields, facilitating convenience in human production and daily life.

Path-planning algorithms include traditional algorithms and intelligent optimization algorithms. Traditional algorithms can be divided into global and local path-planning algorithms according to their basic principles and application areas, among which global path-planning algorithms include Dijkstra’s algorithm [4], the A* algorithm [5], RRT algorithm [6], Probabilistic Roadmaps (PRM) [7], Tabu Search algorithms [8], etc. The A* algorithm is a heuristic search algorithm combining the breadth-first search (BFS) and Dijkstra’s algorithm. RRT and PRM are sampling-based path-planning algorithms suitable for high-dimensional environments and complex terrain, but not for use in narrow and dynamic spaces. Taboo search algorithms are developed based on greedy ideas, and taboos are introduced to solve the problem of local optimization so that they have a strong global search capability. Local path-planning algorithms include the artificial potential field method [9], Dynamic A*(D*)algorithm [10], and Anytime Dynamic A*(AD*) [11]. The artificial potential field method pushes the robot away from the obstacle by artificial force and attracts it to the target location. Potential fields have been applied in mobile robotics [9], but they tend to fall into local minima and perform poorly in narrow regions [12]. Artificial potential fields are used as a steering function in RRT [13] or PRM to improve the efficiency and quality of path search. D* is an improvement based on the A algorithm, particularly suitable for path-planning problems in dynamic environments. Focussed D* [14] and D* Lite [15] are both based on the idea of incremental search of the A* algorithm, which is based on the A* algorithm, by dynamically updating the path cost and the environment model to adapt to changes in the environment. Intelligent optimization algorithms include Genetic Algorithms [16,17], Particle Swarm Optimization [18], Ant Colony Algorithms [19], and path-planning algorithms based on reinforcement learning. The Adaptive Genetic Algorithm [20] is an improvement of the basic genetic algorithm, which greatly improves the convergence accuracy and accelerates the convergence speed. The Niche Genetic Algorithm [21] solves the problem of low population diversity of traditional genetic algorithms. Deep reinforcement learning (DRL) has emerged as a promising approach for path planning in dynamic and uncertain environments. A notable example is the cross-platform deep reinforcement learning model for autonomous navigation, which does not rely on global information and can operate in different scenes. This approach, described in [22], has demonstrated the ability to learn complex behaviors through interaction with the environment and adapt to dynamic obstacles, showing potential for real-time navigation and path planning in environments with unknown or changing conditions.

In the sampling-based algorithm, the RRT* [23] algorithm, an improvement over RRT introduced by Karaman and Frazzoli in 2011 optimizes paths by reconnecting nodes in the tree to reduce the path length and improve quality. The RRT* algorithm can find solutions closer to the optimal path, especially, performing well in large or high-dimensional spaces. RRT Connect [24] is a bidirectional extension of RRT that explores simultaneously from the start and goal points, effectively reducing the time required for path search. RRT*-Smart [25] enhances the path-planning efficiency and optimization capabilities by dynamically adjusting tree structures and introducing heuristic strategies. SRRT* [26] focuses on generating collision-free path-planning solutions in dynamic environments. Sampling needs to be simple but efficient in order to attain a high search speed and quality paths in the search space. The basic RRT algorithm operates as a single-query tree. C. B. Moon and W. Chung [27] introduced the Dual-tree RRT, which flexibly expands nodes within the target workspace. Similarly, C. Long et al. [28] proposed a dual-tree structure, which explores different homotopy paths in each sample. F. Burget et al. [29] proposed a method called Bidirectional Informed RRT*, which is designed to generate asymptotically optimal paths for multiple mobile robots with certain task requirements. Considering the limitations of RRT* in high-dimensional configuration spaces, B. Akgun and M. Stilman [30] introduced sampling bias strategies and node rejection criteria to enhance sampling efficiency.

Recent advancements have focused on adapting RRT-based algorithms to dynamic environments and incorporating kinematic constraints for smoother and more reliable path planning. Notably, Dynamic RRT (D-RRT) [31] has been proposed to handle environments with moving obstacles, offering real-time path replanning by continuously updating the tree structure. Furthermore, a new variant of the RRT algorithm, the Real-Time RRT (RT-RRT) [32], has been developed for fast dynamic environments, where path adaptation is required in response to frequent environmental changes. In addition, research on Clothoid-based path planning has made significant progress, particularly in maintaining smooth and feasible paths under kinematic constraints. Recent studies have proposed integrating Clothoid curves into RRT-based path-planning algorithms to ensure smoothness and prevent jerky movements, which is crucial for robots that need to comply with physical motion limitations [33].

In the real world, robot path planning necessitates achieving path smoothness without collisions with obstacles and avoiding sudden turns or discontinuous movements to enhance robot stability and path-tracking performance. Additionally, it is crucial to ensure that the planned paths adhere to the robot’s dynamic and control constraints to guarantee execution reliability. Path-planning algorithms must possess robustness to generate feasible paths under different environments and uncertain conditions, enabling robots to adapt to complex environments. Kinodynamic RRT [34] extends the original RRT algorithm to effectively plan paths while considering system dynamics and kinematic constraints. Kinodynamic RRT* [35] explores a variant of RRT* specifically designed for optimal motion-planning problems with linear differential constraints. Smooth-RRT [36] aims to minimize the path curvature while generating paths, thereby enhancing robot motion stability and path-tracking accuracy.

To meet the robot’s kinematic constraints and ensure path smoothness, this paper proposes an improved RRT-based algorithm that can quickly plan smooth, obstacle-free paths while adhering to the robot’s kinematic constraints. The goal is to enhance search efficiency and obstacle avoidance capabilities while addressing issues of long computation times and suboptimal paths. Firstly, unlike RRT*, which uses a single-direction search tree, we propose a bidirectional search strategy that expands from both the start and goal nodes simultaneously, improving search efficiency and initially generating a connectable piecewise linear path. Secondly, we use a node reconnection strategy to optimize the initial path, further shortening the total path length and reducing memory usage by nodes. Thirdly, Cubic Spline interpolation is applied to smooth the path while satisfying the robot’s kinematic constraints. We propose a new path deformation strategy to adjust the path: if the smoothed path does not collide with obstacles, no deformation is needed. If a collision occurs, a new Clothoid curve replaces the collision curve to avoid the obstacle. We demonstrate that this method allows the robot to avoid obstacles and generate paths directed towards the goal.

Section 2 provides a detailed description of RRT* and Cubic Spline. Our proposed algorithm is introduced in Section 3, detailing how to generate the initial path, optimize the path through node reconnection, and avoid obstacle collisions during path smoothing. Section 4 presents parameter sets and experimental results comparing our algorithm with others. Section 5 concludes the paper.

## 2. Preliminaries

### 2.1. RRT*

The RRT algorithm sets the start point as the first node of the random tree *T*, and then randomly samples the entire workspace to complete the expansion of the random tree. The nearest node $x_{near}$ to the new node $x_{new}$ in the search tree is found by nearest neighbor search, and the straight line $\left(x_{near},x_{new}\right)$ can be added to the random tree *T* after it passes the collision detection. The above process is repeated until the points in the random tree appear in the range of the goal point $x_{goal}$. Provided there is no collision, the sampling will stop and the goal point will be appended to the random tree *T*. Finally, path backtracking is performed to obtain a feasible path connecting the start point $x_{start}$ and the goal point $x_{goal}$. The new coordinates are given by Equation (1), where δ is the step size:

(1) $$x_{\mathrm{new}}=x_{\mathrm{near}}+\frac{x_{\mathrm{rand}}-x_{\mathrm{near}}}{∥x_{\mathrm{rand}}-x_{\mathrm{near}}∥}\times \delta$$

**Figure 1:**
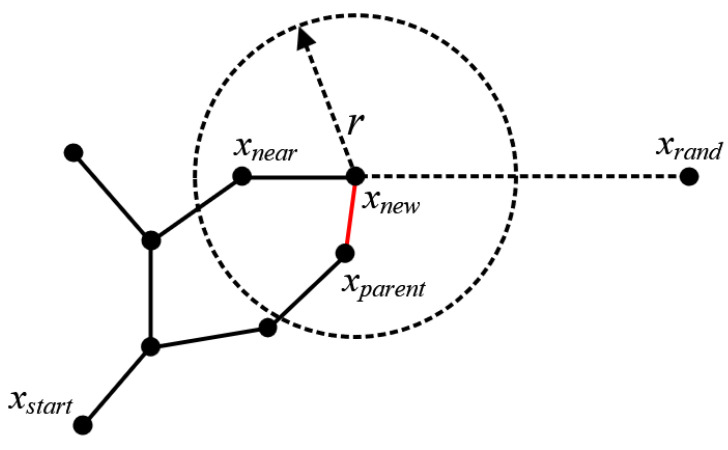
RRT* extension process.

RRT* improves on RRT, mainly by re-selecting the parent nodes and rewiring. As in Figure 1, parent nodes are searched for in the vicinity of the new node $x_{new}$ in a defined radius range *r*. $x_{new}$ is connected to those nodes that can make the distance from $x_{start}$ to $x_{new}$ the shortest and the least costly, and if a more suitable parent node is selected, $x_{new}$ is connected to $x_{parent}$, removing the original connecting line. After the parent node is selected, the nodes are rewired within a certain range near the new node, whether the parent of these nodes is set to $x_{new}$ is checked, and the parent is updated to $x_{new}$ if the cost is reduced.

### 2.2. Cubic Spline

The basic idea of Cubic Spline [37] is to connect given data points in segments and fit them using a cubic polynomial on each subinterval. These subintervals are usually defined by the nodes between the data points. Within each subinterval, the coefficients of the cubic polynomial are defined by an interpolation condition and a smoothing condition. The interpolation condition requires that the curve passes through each data point, while the smoothing condition requires that the curves of neighboring subintervals have the same slope and curvature at the nodes, thus ensuring a smooth curve. Cubic Spline involves fitting a series of cubic polynomials. Suppose there are n + 1 data points, and the 2D spatial coordinates are set as $\left(x_{1},y_{1}\right),\left(x_{2},y_{2}\right),\ldots ,\left(x_{n},y_{n}\right)$, where $x_{0}<x_{1}<\ldots <x_{n}$, and a cubic polynomial is to be fitted between each neighboring data point. Let the cubic polynomial for the ith subinterval be as follows, where $x\in \left[x_{i},x_{i+1}\right]$:

(2) $$S_{i}\left(x\right)=a_{i}+b_{i}\left(x-x_{i}\right)+c_{i}{\left(x-x_{i}\right)}^{2}+d_{i}{\left(x-x_{i}\right)}^{3}$$

where $a_{i},b_{i},c_{i}$, and $d_{i}$ are the coefficients. On each subinterval, the interpolation condition and the smoothing condition need to be satisfied. The interpolation condition requires that the curve passes through each data point with the following conditions:

(3) $$S_{i}\left(x_{i}\right)=y_{i},\mathrm{for}\;i=0,1,\ldots ,n-1$$

On the other hand, the smoothing condition requires that the curves of neighboring subintervals have the same slope and curvature at the nodes, i.e., $S^{\prime }\left(x\right)$ and $S^{\prime \prime }\left(x\right)$ are consecutive on the interval [$x_{0},x_{n}$], where *i* = 0, 1,…, n−2:

(4) $$\begin{array}{c} S_{i}^{\prime }\left(x_{i+1}\right)=S_{i+1}^{\prime }\left(x_{i+1}\right)\;and\;S_{i}^{\prime \prime }\left(x_{i+1}\right)=S_{i+1}^{\prime \prime }\left(x_{i+1}\right) \end{array}$$

Suppose the second derivative is 0 at both endpoints:

(5) $$\begin{array}{c} S_{0}^{\prime \prime }\left(x_{0}\right)=0\;\mathrm{and}\;S_{n-1}^{\prime \prime }\left(x_{n}\right)=0 \end{array}$$

Based on these interpolation and smoothing conditions, the formula for the coefficients $a_{i},b_{i},c_{i}$, and $d_{i}$ can be obtained.

(6)gi=xi+1−xi,∀i=0,1,…,n−1(7)Si(xi)=ai(8)Si+1(xi+1)=ai+1=ai+bigi+ci(gi)2+di(gi)3(9)Si′(xi)=bi,alsobi+1=bi+2cigi+3di(gi)2(10)Si″(xi)=2ci,alsoci+1=ci+3digi

Finally, the coefficients $a_{i},b_{i},c_{i}$, and $d_{i}$ can be obtained:

(11)ai=Si(xi)(12)bi=ai+1−aigi−gi(2ci+ci+1)3(13)cicanbesolvedinAc=B(14)di=ci+1−ci3gi

In order to make the robot conform to the kinematic constraints, we introduce constraints to ensure that paths can be planned while satisfying the steering angular velocity constraints. On the path, the steering angular velocity is obtained indirectly by calculating the curvature of the path. Assuming that the linear velocity of the robot is *v*, the steering angular velocity can be expressed as:

(15) $$\begin{array}{c} \omega =\frac{v}{R} \end{array}$$

where *R* is the radius of curvature of the path, and the curvature of the path is related to the second-order derivative (acceleration) of the Cubic Spline. The curvature is obtained by taking the second-order derivative of the Cubic Spline:

(16) $$\begin{array}{cc} k(s) & =\frac{x^{\prime \prime }\left(s\right)y^{\prime }\left(s\right)-y^{\prime \prime }\left(s\right)x^{\prime }\left(s\right)}{{\left(x^{\prime }{\left(s\right)}^{2}+y^{\prime }{\left(s\right)}^{2}\right)}^{3/2}} \end{array}$$

where *s* is the arc length of the curve, x(s) and y(s) are the parameterized coordinates of the path, respectively, $x^{\prime }\left(s\right)$ and $y^{\prime }\left(s\right)$ are their derivatives, and $x^{\prime \prime }\left(s\right)$ and $y^{\prime \prime }\left(s\right)$ are second-order derivatives. The curvature of the path *k* should always be less than or equal to this maximum value $k_{max}$:

(17) $$\begin{array}{c} k_{max}=\frac{\omega_{max}}{v} \end{array}$$

The optimization objective of Cubic Spline is to make paths smooth and pass through specified start and end points while avoiding constraints such as collisions. The basic objective function for minimizing the smoothness of the path is as follows:

(18) $$\begin{array}{c} J_{path}=\int_{s_{0}}^{s_{1}}\left({\left(x^{\prime \prime }\left(s\right)\right)}^{2}+{\left(y^{\prime \prime }\left(s\right)\right)}^{2}\right)ds \end{array}$$

We add a penalty term to the existing objective function to control the curvature of the path from exceeding the maximum limit $k_{max}$:

(19) $$\begin{array}{c} J_{total}=J_{path}+\lambda \int_{s_{0}}^{s_{1}}max{\left(0,k\left(s\right)-k_{max}\right)}^{2}ds \end{array}$$

where λ is the weight coefficient of the penalty term, which is used to regulate the trade-off between the curvature constraints and smoothness. We optimize the objective function by adding constraints and penalty terms to the curvature, thus finally ensuring that the path satisfies the kinematic constraints of the robot.

## 3. Proposed Algorithm

In this section, we detail an improved algorithm based on RRT* as a way to realize that the robot can find an efficient and smooth path under the kinematic constraints. The specific steps of our algorithm are as follows:

1. Based on RRT*, we use the bidirectional RRT* algorithm to simultaneously extend two trees starting at an initial point and a goal point to quickly find an initial simple path.
2. The initial path is optimized using a node reconnection strategy to reduce the number of tree nodes and shorten their path lengths.
3. The optimized path generated by (2) is smoothed based on Cubic Spline interpolation, where a new path deformation strategy is used to avoid obstacle collisions, and finally, a curved path is generated suitable for the robot.

Algorithm 1 shows the overall steps of our algorithm. The BuildaPath function is implemented to generate the initialpath, the NodeReconnection function optimizes the initialpath to generate the optimizedpath, and finally, the optimizedpath is smoothed by SmoothPath to generate finalpath. If no connectable path is found, the algorithm replans.
**Algorithm 1** Path Planning Based on RRT* 1:initialpath←⌀ 2:i← 0 3:**while** True **do** 4:    i←i+1 5:    initialpath← BuildaPath(map$,x_{start},x_{goal}$) 6:    optimizedpath← NodeReconnection(map,initialpath) 7:    finalpath← SmoothPath(map,$optimizedpath,x_{goal}$) 8:    **if** path is Null **then** 9:        Continue10:    **else**11:        Break12:    **end if**13:**end while**14:**return** path

### 3.1. Improved Bidirectional RRT*

In the RRT* algorithm, only one random search tree grows from the starting point, because of the randomness of the sampling point generation, the problem of slower convergence occurs. Bi-RRT* constructs two trees $T_{a}$ and $T_{b}$ from the starting point and the goal point at the same time, and randomly generates $x_{rand}$ as the target guide in the search space to expand $T_{a}$ and $T_{b}$ alternately after collision detection, and adds a new node $x_{new}$ to $T_{a}$ or $T_{b}$ and outputs a connected path if the distance between $T_{a}$ and $T_{b}$ is less than the threshold, respectively. Node $x_{new}$ is added to $T_{a}$ or $T_{b}$ and then the process of re-selecting the parent node and rewiring are executed, respectively, and the connected path is output if the distance between $T_{a}$ and $T_{b}$ is less than a threshold value. Based on this, we improve the expansion targets of the two trees. Unlike Bi-RRT*, where both trees use random points for expansion, we use the target point $x_{goal}$ for $T_{a}$ to reduce unnecessary expansion, while $T_{b}$ expands towards the last node of $T_{a}$ to accelerate path connectivity. If an obstacle is encountered while generating a new node, the node closest to the target point is selected from the tree, and a new expansion target is generated by random sampling in its neighborhood. The overall steps are shown in Algorithm 2, and the specific expansion process of the tree is detailed in Algorithm 3.

**Figure 2:**
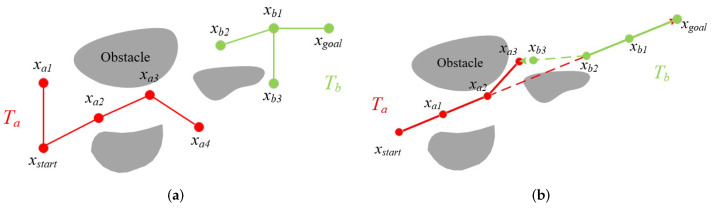
Tree-generation process for Bi-RRT* (**a**) and the improved Bi-RRT* (**b**). (**a**) Bi-RRT*: $T_{a},T_{b}$ generates new nodes by random sampling. (**b**) The improved Bi-RRT*: $T_{a},T_{b}$ expands to generate new nodes with different goals.

Figure 2 demonstrates the difference between the improved Bi-RRT* and Bi-RRT* in terms of the expansion process. Figure 2a shows the process of generating a randomized tree from Bi-RRT*, and Figure 2b shows the improved Bi-RRT*. As shown in Figure 2b, $T_{a}$ generates $x_{a1}$ towards $x_{goal}$ by a certain step size, $T_{b}$ generates $x_{b1}$ towards the current last node $x_{a1}$ of $T_{a}$, followed by the same generation of $x_{a2}$, $x_{b2}$. When an obstacle is detected while $x_{a2}$ is expanding towards $x_{goal}$, it is randomly sampled in the around of $x_{a2}$ to generate a new node $x_{a3}$, and $x_{b2}$ expands towards the last node of $T_{a}$ at this time, $x_{a3}$, to generate $x_{b3}$.
**Algorithm 2** Generate a Simple Path 1:**function** BuildaPath(map$,x_{start},x_{goal}$) 2:    i← 0 3:    $T_{a},T_{b}\leftarrow ⌀$ 4:    $T_{a}.$add$\left(x_{start}\right)$ 5:    $T_{b}.$add$\left(x_{goal}\right)$ 6:    isconnect= False 7:    **while** isconnect== False and i<N **do** 8:        i←i+1 9:        $T_{a}\leftarrow$ Extend $\left(T_{a},x_{goal}\right)$10:        $T_{b}\leftarrow$ Extend $(T_{b},$ End$\left(T_{a})\right)$11:        **if** Connect$(T_{a},T_{b})==$ True **then**12:           isconnect= True13:           path← FindPath$\left(T_{a},T_{b}\right)$14:        **end if**15:    **end while**16:    **return** path17:**end function**
**Algorithm 3** Tree Extension Strategy 1:**function** Extend($T,x_{target}$) 2:    $x_{nearest}\leftarrow$NearestNode$\left(T,x_{target}\right)$ 3:    $x_{new}\leftarrow$ Steer$\left(x_{nearest},x_{target}\right)$ 4:    **if** Collision$(x_{nearest},x_{new})==$False **then** 5:        $V\leftarrow V\cup \{x_{new}\}$ 6:        $E\leftarrow E\cup \{x_{nearest},x_{new}\}$ 7:    **else** 8:        $x_{rand}\leftarrow$ Nearsample$\left(T,x_{nearest}\right)$ 9:        $x_{new}\leftarrow$ Steer$\left(x_{nearest},x_{rand}\right)$10:        **if** Collision$(x_{nearest},x_{new})==$False **then**11:           $X_{near}\leftarrow$ Near($T,x_{new}$)12:           $x_{parent}\leftarrow$ ChooseParent$\left(X_{near},x_{nearest},x_{new}\right)$13:           $V\leftarrow V\cup \{x_{new}\}$14:           $E\leftarrow E\cup \{x_{nearest},x_{new}\}$15:           T← Rewire$\left(T,X_{near},x_{new}\right)$16:        **end if**17:    **end if**18:    **return** T←{V,E}19:**end function**

### 3.2. Node Reconnection Strategy

Since the path generated by the improved Bi-RRT* is not smooth, it needs optimization to reduce memory usage and improve the efficiency of subsequent smoothing. The key point kp is defined. The starting point is taken as the first key point, and collision detection is performed on the nodes along the path. If the line between kp and the current node does not collide with the obstacle, the next node is checked. Until a collision occurs, the previous node of the collision node is taken as the new key point and added to the key point sequence. Collision detection is performed on the new key point with the subsequent node, and the above steps are repeated until the target point is reached with no collision, at which point the target point is added to the sequence. The key point sequence represents the optimized path.

**Figure 3:**
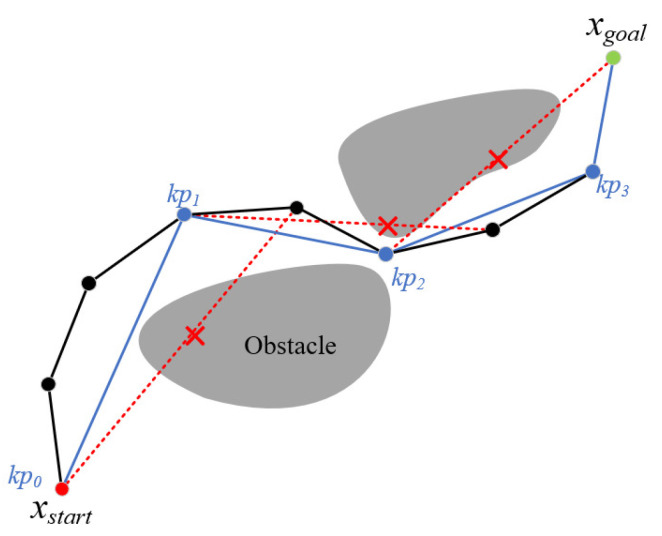
Path node reconnection process. kp is the key point, the black line is the initial path, and the blue line is the optimized path after node reconnection.

Figure 3 illustrates the process of node reconnection, where the black solid line is the initial path and the blue solid line is the optimized path after node reconnection. We take the starting point $x_{start}$ as the first key point $kp_{0}$ (abbreviated as kp in the figure) and sequentially detect whether $kp_{0}$ collides with the nodes on the path, and if it collides, the previous node of the colliding node is taken as the second key point $kp_{1}$. The above process is repeated until $kp_{3}$ is detected to have no collision with the goal point $x_{goal}$, and the sequence consisting of kp is the optimized path. In Algorithm 4, all key points are found by iteration (lines 5–15) and added to the sequence of keypoints. Collision detection is performed using the Collision function (line 6) and the loop is interrupted when $x_{goal}$ is detected. The keypoints sequence is the optimized path.
**Algorithm 4** Optimized Path 1:**function** NodeReconnection(map, $x_{start}$, $x_{goal}$) 2:    $keypoints\leftarrow \{x_{start}\}$ 3:    $kp\leftarrow x_{start}$ 4:    i← 0 5:    **while** true **do** 6:        **while** Collision(kp, $path_{i}$, map) == False **do** 7:           **if** $path_{i}==x_{goal}$ **then** 8:               $keypoints\leftarrow keypoints\cup path_{i}$ 9:               Break10:           **end if**11:           i←i+112:        **end while**13:        $kp\leftarrow path_{i-1}$14:        keypoints←keypoints∪kp15:    **end while**16:    **return** keypoints17:**end function**

### 3.3. Path Smoothing and Obstacle Avoidance

To generate paths that satisfy the robot’s kinematic constraints, the optimized paths need to be smoothed. The optimized path is collision-free, but when smoothed using methods such as curve interpolation [38,39], the motion may deviate from the original path, potentially causing collisions in complex maps.To solve this problem, we propose a path deformation strategy that aims to modify the path to avoid obstacles during smoothing. The advantage of the Clothoid curve is that the curvature varies continuously, which enables the avoidance of obstacles and compliance with the kinematic constraints of the robot’s motion. Additionally, the centrifugal acceleration is continuous, preventing sudden changes that could otherwise impair the robot’s stability. The optimized path is smoothed using Cubic Spline, and a collision check is performed to ascertain whether the path collides with any obstacles. If a path from a node collides with an obstacle, we generate a series of Clothoid curves with curvature *k* and arc length *l* beginning from the node. The curve that does not collide with obstacles and whose end node is closest to the goal point is chosen to replace the original curve segment. The Euclidean distance is used to calculate the distance between nodes. This process completes the path deformation.

In the Clothoid curve, we define the curvature as positive if the rotational change of the tangent vector is clockwise. For a given pose $\left(x_{0},y_{0},\theta_{0}\right)$ and curvature *k* and arc length *l*, where $x_{0},y_{0}$ are the two-dimensional coordinates of the given pose and $\theta_{0}$ is the angle between the x-axis and the pose direction. We define (x(s),y(s)) to denote the x-axis coordinates and y-axis coordinates at the arc length *s*:

(20) $$\begin{array}{cc} x(s) & =\sqrt{\frac{2}{k}}\cdot C\left(\sqrt{\frac{k}{2}}\cdot s\right) \end{array}$$

(21) $$\begin{array}{cc} y(s) & =\sqrt{\frac{2}{k}}\cdot S\left(\sqrt{\frac{k}{2}}\cdot s\right) \end{array}$$

where C(z) and S(z) are the Fresnel cosine integral and sine integral:

(22) $$\begin{array}{cc} \; & C\left(z\right)=\int_{0}^{z}\cos\left(\frac{\pi }{2}u^{2}\right)du \end{array}$$

(23) $$\begin{array}{cc} \; & S\left(z\right)=\int_{0}^{z}\sin\left(\frac{\pi }{2}u^{2}\right)du \end{array}$$

The coordinates of points (x,y,θ) on the Clothoid curve are as follows:

(24)x=x0+cos(θ0)·x(s)−sin(θ0)·y(s)(25)y=y0+sin(θ0)·x(s)+cos(θ0)·y(s)(26)θ=θ0+k·s

**Figure 4:**
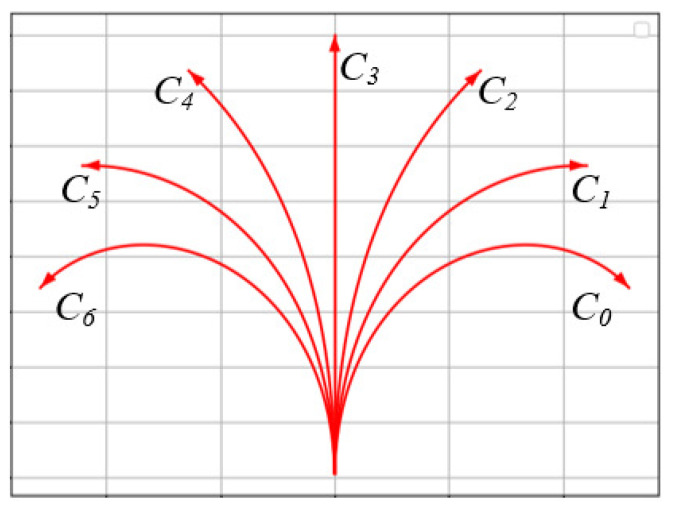
Different curvatures of the Clothoid curve. $C_{0}$, $C_{1}$,…,$C_{6}$ represent curvature = −0.3, −0.2, −0.1, 0, 0.1, 0.2, 0.3, respectively.

As shown in Figure 4, a Clothoid curve is formed from the position (0,0,π/2) with *l* = 15 and *K* = −0.3, −0.2, −0.1, 0, 0.1, 0.2, 0.3.

**Figure 5:**
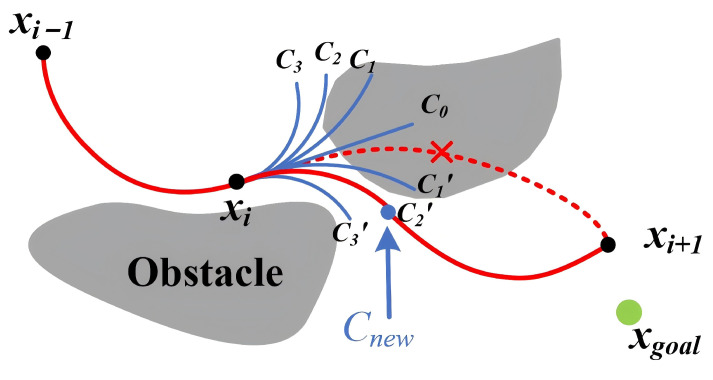
Obstacle avoidance strategy during path smoothing.

Algorithm 5 gives the strategy for path generation and path deformation. First, the finalpath set spath is initialized as empty. Lines 5–23 smooth the optimized path. We use CubicSpline to generate a path (line 6), and when colliding with an obstacle, as shown by the red dashed line in Figure 5, the Clothoidcurve function generates ||K|| Clothoid curves with a given curvature *k* and arc length *l* (line 11). From among the curves that pass the obstacle detection, we select the curve $c_{new}$ closest to the goal point $x_{goal}$ as the new path replacing the original path (lines 12–20) and then continue the path generation from the end of the curve.
**Algorithm 5** Path Smooth and Collision Avoidance 1:**function** SmoothPath(map$,path,x_{goal},K$) 2:    $d_{min}\leftarrow \infty$ 3:    spath←⌀ 4:    spoints←⌀ 5:    **for** i=0…|path|−1 **do** 6:        spoints← CubicSpline$\left(path_{i}\right)$ 7:        **if** CheckPath(spoints)== no collision **then** 8:           spath←spath∪{spoints} 9:        **else**10:           **for** *k* in *K* **do**11:               c= Clothoidcurve$\left(path_{i},l,k\right)$12:               **if** Checkpath(c)== no collision **then**13:                   d=|| Endpoint$\left(c\right)-x_{goal}$||14:                   **if** $d<d_{min}$ **then**15:                       $d_{min}=d$16:                       $c_{new}=c$17:                   **end if**18:               **end if**19:           **end for**20:           $spath\leftarrow spath\cup \{c_{new}\}$21:           $path_{i+1}\leftarrow$ Endpoint$\left(c_{new}\right)$22:        **end if**23:    **end for**24:    **return** spath25:**end function**

### 3.4. Time Complexity Analysis

Our algorithm consists of three steps: initial path generation, node reconnection, and path smoothing. We use the improved bi-RRT* to generate initial paths with a total time complexity of:

(27) $$\begin{array}{c} T_{initialpath}=O\left(nlogn\right)+O\left(nc\right) \end{array}$$

where random sampling generates *n* nodes with a time complexity of O(n) and nearest neighbor search with a time complexity of O(nlogn). Assuming that the time complexity of each collision detection is O(c), which depends on the number and complexity of obstacles, the time complexity of collision detection is O(nc). Traversing all nodes along the initial path and detecting keypoints during node reconnection, assuming the number of nodes on the initial path is *d* (usually d≪n) and the number of keypoints is *m*, the time complexity is:

(28) $$\begin{array}{c} T_{nodereconnection}=O\left(d\right)+O\left(mc\right) \end{array}$$

In the worst case, the number of keypoints *m* is the same as the number of nodes *d* on the initial path, so the time complexity can be approximated as:

(29) $$\begin{array}{c} T_{nodereconnection}=O\left(d^{2}\cdot c\right) \end{array}$$

However, in the general case, usually the number of keypoints is significantly lower than the number of nodes on the initial path, so the complexity is closer to O(d) or O(d·c).

We use Cubic Spline to curve-fit the path after nodereconnection and perform collision detection. If a collision occurs, the corresponding segment is replaced with a Clothoid curve. Assuming that the number of collisions is $h_{p}$ and the time complexity of using Clothoid curve replacement is O(l) each time, the time complexity is:

(30) $$\begin{array}{c} T_{pathsmooth}=O\left(m\right)+O\left(mc\right)+O\left(h_{p}\cdot l\right) \end{array}$$

In summary, since $m,d,h_{p}\ll n$ and assuming that c,l are constants, the total time complexity is:

(31)Ttotal=Tinitialpath+Tnodereconnection+Tpathsmooth(32)  =O(nlogn)+O(nc)

The total time complexity of the algorithm mainly depends on the node sampling and collision detection in the initial path-generation phase, and the complexity of node reconnection and path optimization is relatively low, but the time complexity of the optimization phase may increase significantly when the obstacles are dense and the number of collisions is high.

## 4. Experimentation

We conduct experiments to evaluate the performance of the proposed algorithm. For this experiment, our proposed algorithm is compared with RRT* with Bspline(RB) and Kinodynamic RRT*(Kino-RRT*) to evaluate their performance.

**Figure 6:**
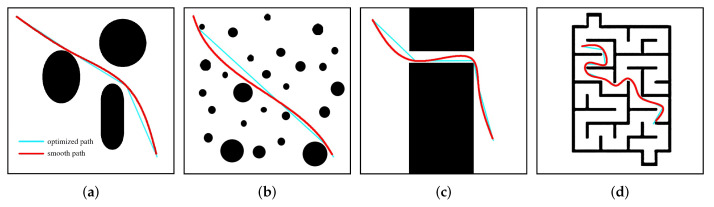
(**a**) Large obstacle, (**b**) multi-obstacle, (**c**) narrow passage, (**d**) complex maze.

We evaluated the performance of the path planner and path generation strategy in four environments (see Figure 6). These four maps, each 500 × 500 in size, represent a large obstacle environment, a multi-obstacle environment, a narrow-passage environment, and a complex-maze environment, providing varied environments for evaluation. Different environments are beneficial for testing the superiority of our algorithm’s performance. We set several metrics to assess the algorithm’s performance, such as the time cost for generating the final path, the number of path nodes, and the path length. Additionally, we evaluated the path smoothness by calculating the average curvature change rate of the entire path. Each metric was averaged over 50 runs in each environment. All experiments are conducted on a PC with 3.00 GHz Intel Core i5 and 8 GB of RAM.

### 4.1. Comparison of Initial Path and Optimized Path

**Figure 7:**
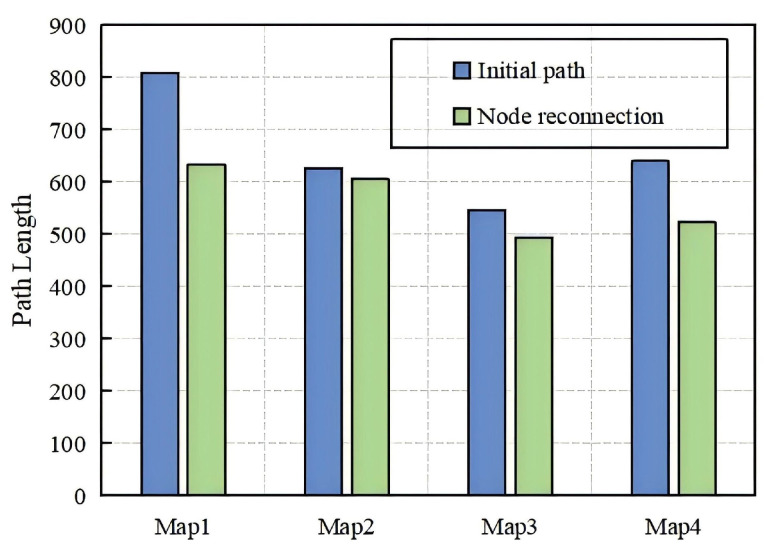
Comparison of path lengths for initial paths and after node reconnection in four maps.

The path node reconnection strategy reduces the number of path nodes, shortens the path length, and reduces the memory footprint. We perform the algorithm 50 times in each of the four environments to obtain the path length before and after node reconnection. In Figure 7, blue color represents the initial path length and the green color represents the path length after node reconnection. The results show that the path length after the node reconnection strategy is shortened by 3–21% in either environment.

### 4.2. Curvature *K* and arc Length *l*

In the path deformation strategy (Section 3), the parameter *k* plays a crucial role as it controls the degree of path deformation. Setting *k* appropriately is essential because it influences how much the path deviates from its original shape. A larger *k* tends to increase the path length, which may not favor optimal path generation. If the value of *k* is small, it is not easy to cross the obstacles. The parameter *K* represents the curvature set, and we try to set the *K* to be as reasonable as possible and consistent with the kinematic constraints of the robot. Finally, we set k=1, which means that the curvature set *K* ranges from −1 to 1 in steps of 0.1, forming *K* = {−1, −0.9, −0.8, …, 0.8, 0.9, 1}.

The ClothoidCurve function contains the arc length *l* that controls the length of the generated Clothoid curve. Larger *l* will deform excessively, thus affecting the generation of optimal paths, while too small *l* increases the memory footprint as well as increasing the computational complexity, so it is crucial to evaluate the impact of the *l* value on the efficiency of the algorithm. To achieve this, we set *l* in the range of [5,17], with an interval of 2. We count the path lengths of our algorithm in four environments with different values of *l* so that *l* can be adjusted to generate more optimal paths. The results are presented in Table 1. Comparative analysis indicates that Map 1 and Map 3 work best at *l* = 15 and Map 2 and Map 4 work best at *l* = 7 because it is easier to use long arcs for substitution in simpler environments, and shorter arcs work better in complex environments. In the end, we set *l* to 15 in Map 1 and Map 3, and 7 in Map 2 and Map 4.

Adjusting *k* and *l* based on the complexity of the map helps in generating more optimal paths that adhere to the robot’s kinematic constraints and efficiently navigate through various environments.

### 4.3. Computational Time Trend Analysis

**Figure 8:**
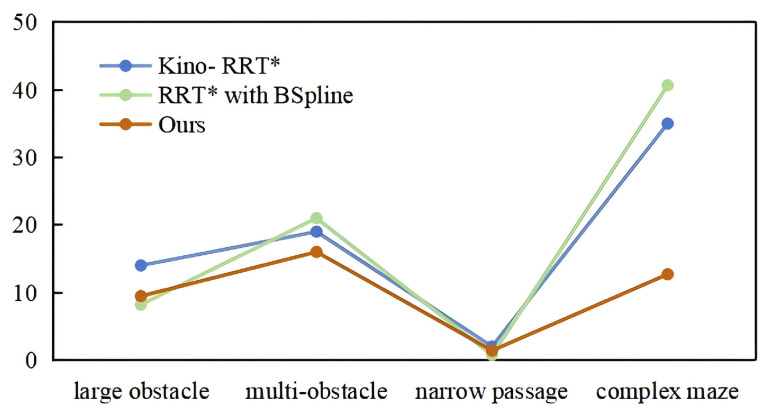
Comparison of computational time trend between Kino-RRT*, RB, and ours in four maps.

We compared the average running time of our algorithm with Kino-RRT* and RB for generating final paths in four maps. As shown in Figure 8, the line graphs show the computational time trends of the three algorithms in the four environments, with the horizontal axis denoting the maps, the vertical axis denoting the average running time (in seconds), and the three lines denoting the computational time of Kino-RRT*, RB, and our algorithm, respectively.

It can be seen from the graphs that in more complex maps, such as the multi-obstacle and complex maze, the three algorithms spend more time. The trend of the running time of our algorithm is smoother. In the complex maze, it can be clearly seen that the running time is lower than the other algorithms, which may be due to the improved computational efficiency through purposeful node sampling and reduction of redundant nodes in the initial path-generation and node-reconnection phases. However, RB spends comparable or even slightly better time than our algorithm in the large-obstacle environment. This is because in the simple environment, RB does not need path optimization, and node reconnection and path optimization bring extra overhead. In summary, our algorithm shows a significant efficiency advantage in complex environments (e.g., multi-obstacle and complex maze), while it performs similarly to the control algorithm in simple environments. The experimental results validate the effectiveness of the improved algorithm in dealing with complex path-planning problems.

### 4.4. Large Obstacle Environment

**Figure 9:**
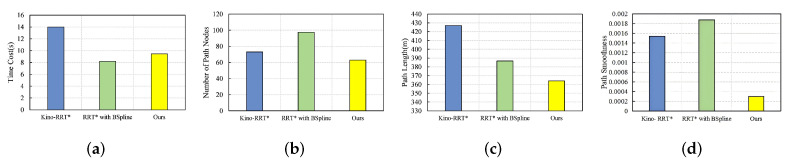
Comparison of three algorithms in large obstacle environment under four metrics: (**a**) time cost, (**b**) number of path nodes, (**c**) path length, (**d**) path smoothness.

In the large obstacle environment, the start point is located at (10, 10), and the goal point is located at (450, 450), as shown in Figure 6a. The performance of the four algorithms in the large obstacle environment is given in Figure 9. From Figure 9a, it can be seen that our algorithm is higher than RB and lower than Kino-RRT* in terms of time cost, maybe because we use a bidirectional search strategy in generating the initial paths to reduce the time but use a path deformation strategy in the path-smoothing phase to increase the computation time. Our algorithm outperforms the remaining two algorithms in terms of the number of nodes and path length, mainly because we optimize the paths using a node reconnection strategy to shorten the path length. The curvature of a curve is the rate of rotation of the tangent direction angle to the arc length for a point on the curve, indicating how much the curve bends at a given point, so the smaller the curvature, the less the curve bends. In terms of path smoothness, we have the smallest value, followed by Kino-RRT*, RB, which proves the smoothness of our path and that it is more suitable for robot walking.

### 4.5. Multi-Obstacle Environment

**Figure 10:**
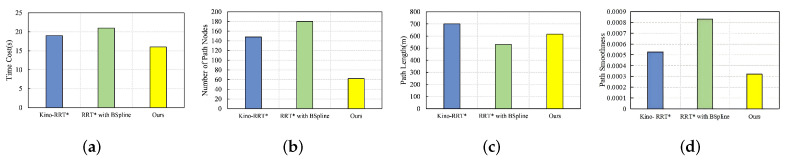
Comparison of three algorithms in multi-obstacle environment under four metrics: (**a**) time cost, (**b**) number of path nodes, (**c**) path length, (**d**) path smoothness.

In the multi-obstacle environment, the start point is located at (10, 10), and the goal point is located at (450, 450), as shown in Figure 6b. Figure 10 compares the performance of the four algorithms in the multi-obstacle environment. Our algorithm does not differ much from the other two algorithms in terms of time cost, but the number of path nodes is smaller than the remaining two algorithms by more than 50%. It is about 20% higher than RB in terms of path length, mainly due to the higher probability of detecting collisions in environments with more obstacles, and thus more costly obstacle avoidance using the Clothoid curve. It still outperforms the remaining two algorithms in terms of path smoothing.

### 4.6. Narrow Passage

**Figure 11:**
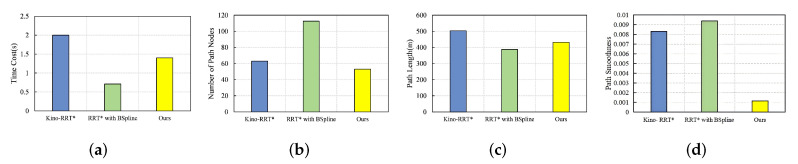
Comparison of three algorithms in narrow passage environment under four metrics: (**a**) time cost, (**b**) number of path nodes, (**c**) path length, (**d**) path smoothness.

In the narrow passage environment, the start point is located at (20, 60), and the goal point is located at (400, 300), as shown in Figure 6c. The performance of the three algorithms is illustrated in Figure 11. The path lengths of Kino-RRT* and our algorithm are longer due to the consideration of kinematic constraints. The search space is more complete in the narrow passage environment, and the bidirectional search tree strategy with node reconnection greatly reduces the number of path nodes compared to the full graph random search strategy of RRT*. Our algorithm is considerably lower than the other two algorithms in terms of path smoothness.

### 4.7. Complex Maze

**Figure 12:**
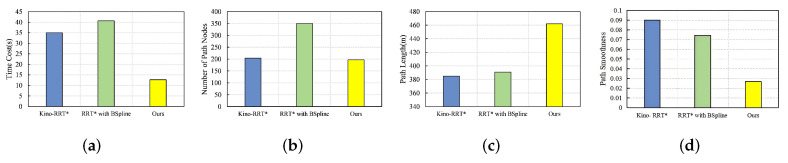
Comparison of three algorithms in complex maze environment under four metrics in (**a**) time cost, (**b**) number of path nodes, (**c**) path length, (**d**) path smoothness.

In the narrow passage environment, the start point is located at (120, 100), and the goal point is located at (300, 270), as shown in Figure 6d. The performance of the three algorithms is illustrated in Figure 12. Unlike our algorithm, other algorithms randomly generate many useless nodes in the tree generation phase; in particular, the search space of RRT* covers the whole planning space, which leads to low node utilization and slow path planning. In contrast, our algorithm adopts a bidirectional search tree strategy with goal orientation during tree expansion, which reduces the number of nodes per tree, improves the efficiency of nearest-neighbor search and the probability of obtaining valid samples, speeds up initial path generation, and boasts excellent performance in complex maze environments.

Our algorithm has much lower time cost than the other two algorithms, and the number of path nodes is about 40% less than RB. In the complex maze environment, the initial path is more twisted, which leads to the need to use the path deformation strategy several times for obstacle avoidance in the path-smoothing phase, and finally, causes the final path length to be 17–20% higher than that of the other two algorithms. The lower the path smoothness means the smoother the paths are, and our algorithm performs better.

## 5. Discussion

In this study, we proposed a robot path-planning algorithm based on RRT*, addressing issues such as long computation times, suboptimal paths, and low path smoothness success rates that plagued previous algorithms. The algorithm improves search efficiency and obstacle avoidance. In this algorithm, the bi-directional expansion strategy with goal orientation shortens the time to generate initial paths and reduces the number of nodes in the expanded tree. The node reconnection strategy optimizes both the length of the initial path and the number of nodes. We introduced a novel obstacle avoidance strategy, the path deformation strategy, which adjusts local paths while ensuring collision-free navigation in complex environments. Experimental validation demonstrated that our proposed algorithm outperforms previous algorithms in terms of time cost, path length, and path smoothness, while generating smooth paths for robot navigation.

On the one hand, the proposed algorithm improves path-planning efficiency. On the other hand, our algorithm has limitations, particularly in adapting to dynamic environments. Future research should focus on enhancing adaptability in dynamic environments by integrating real-time perception and dynamic path replanning mechanisms to handle moving obstacles or newly appearing obstacles. Further optimization is also needed to improve real-time performance and computational efficiency, especially for path planning in large-scale or high-dimensional environments.

## Figures and Tables

**Table 1:** The average path length of our algorithm in four environments with different values of the parameter *l*.

Map	*l*						
	5	7	9	11	13	15	17
Map 1	632.4	632.7	629.5	630.8	629.1	**625.3**	641.4
Map 2	604.8	**603.8**	613.7	618.2	614.1	621.3	647.3
Map 3	502.2	499.6	525.3	499.1	511.4	**487.6**	510.9
Map 4	423.9	**417.2**	456.5	426.4	427.7	427.5	447.1

## Data Availability

The original contributions presented in the study are included in the article. Further inquiries can be directed to the corresponding author.

## Abbreviations

The following abbreviations are used in this manuscript:

RRT	Rapidly-exploring Random Trees
D*	Dynamic A*
